# Prediction of Compressive Strength of Concrete Using Explainable Machine Learning Models

**DOI:** 10.3390/ma18215009

**Published:** 2025-11-03

**Authors:** Hainan Fu, Xiong Zhou, Pengfei Xu, Dandan Sun

**Affiliations:** 1 Shield and Underground Engineering Research Institute, China Railway 15th Bureau Group Co., Ltd., Shanghai 200070, China; fuhainan.15g@crcc.cn (H.F.); xupengfei@email.cugb.edu.cn (P.X.); 2 School of Engineering and Technology, China University of Geosciences, Beijing 100083, China; zhouxiong@cugb.edu.cn

**Keywords:** concrete compressive strength, machine learning, Bayesian optimization, SHAP analysis, mix proportion optimization

## Abstract

Predicting the compressive strength of concrete is essential for engineering design and quality assurance. Traditional empirical formulas often fall short in capturing complex multi-factor interactions and nonlinear relationships. This study employs an interpretable machine learning framework using Gradient Boosting Trees, Random Forest, and Backpropagation Neural Networks to predict concrete compressive strength. Bayesian optimization was employed for hyperparameter tuning, and SHAP analysis was used to quantify feature contributions. Based on 223 sets of compression test data, this study systematically compared the predictive performance of the five models. Results demonstrate that the CatBoost model achieved the best results, R^2^ of 0.9388, RMSE of 2.7131 MPa, and MAPE of 5.45%, outperforming other models. SHAP analysis indicated that cement content had the greatest impact on strength, followed by water content, water reducer, fly ash, and aggregates, with notable interactive effects between factors. Compared to the empirical formula in the current industry standard Specification for Mix Proportion Design of Ordinary Concrete, the CatBoost model showed higher accuracy under specific raw material and curing conditions, with MAPE values of 2.94% and 5.96%, respectively. The optimized CatBoost model, combined with interpretability analysis, offers a data-driven tool for concrete mix optimization, balancing high precision with practical engineering applicability.

## 1. Introduction

Concrete, as a key material in modern construction engineering, exhibits compressive strength that serves as a critical indicator determining structural safety and durability [1]. Traditional strength prediction methods predominantly rely on empirical formulas [2,3,4] or laboratory tests of concrete specimens [5,6,7]. The former is limited by simplified mathematical assumptions that fail to capture the material’s nonlinear characteristics, while the latter suffers from lengthy cycles, high costs, and an inability to provide real-time guidance during construction. With the rapid advancement of machine learning techniques, data-driven predictive models for strength have gradually become a focus of research. These models can capture complex feature relationships from historical mix proportion data, significantly enhancing prediction efficiency [8,9,10,11].

In recent years, ensemble learning algorithms (such as Gradient Boosting Trees and Random Forest) and neural networks have demonstrated excellent performance in applications within the construction engineering field. Gradient Boosting Trees (GBT), as a representative ensemble learning algorithm, iteratively constructs a sequence of weak learners to optimize the model [12,13,14]. This approach offers advantages in efficiently handling structured data, memory optimization, and processing categorical features. Random Forest (RF) enhances model generalization by constructing multiple decision trees and aggregating their results [15]. It strengthens model robustness through twofold randomization (bootstrap sampling and feature subset selection) and supports feature importance evaluation. Backpropagation Neural Networks (BPNN) achieve complex function approximation via nonlinear combinations of multi-layer artificial neurons, operating through two core phases (forward propagation and error backpropagation) where continuous updates to neuronal weights and thresholds reduce model fitting errors within target ranges to improve prediction accuracy [16,17].

Existing research has extensively validated the effectiveness of various algorithms in predicting concrete strength: Elshaarawy et al. [18] employed both ensemble and non-ensemble machine learning models for concrete strength prediction, demonstrating that CatBoost delivers superior predictive performance with significantly higher accuracy than traditional regression methods. Sun et al. [19] and Wu et al. [20] effectively utilized Backpropagation Neural Networks to predict concrete compressive strength. Meddage et al. [21] and Abdellatief et al. [22,23] accurately predicted concrete compressive strength using gradient boosting algorithms (XGBoost). Kashem et al. [24] introduced Light Gradient Boosting Machine (LGBM), Extreme Gradient Boosting (XGBoost), Random Forest (RF), and hybrid machine learning (HML) approaches to predict the compressive strength of rice husk ash concrete, revealing that the hybrid XGBoost-LGBM model exhibits outstanding predictive and generalization capabilities. Additionally, Alyami et al. [25] applied seven machine learning algorithms to predict the compressive strength of fiber-reinforced concrete (3DP-FRC), finding that the Gene Expression Programming (GEP) model achieved higher accuracy in the validation set. Sami et al. [26] collected 8 sets of high-strength concrete data samples from literature to develop predictive models for tensile and compressive strength, proving that the Gaussian Process Regression (GPR) model delivers greater accuracy in predicting both tensile and compressive strength of concrete. It is noteworthy that Abbas et al. [27] utilized a deep autoencoder for damage identification in underground metro shield tunnels, demonstrating the potential of deep learning in structural health monitoring. Concurrently, Kumar et al. [28] systematically reviewed research trends and future directions of machine and deep learning in concrete strength prediction, providing theoretical support for this study.

While machine learning algorithms demonstrate significant advantages in concrete strength prediction, they face challenges in quantifying feature contributions and interaction effects, resulting in a lack of theoretical guidance for concrete mix proportion optimization; particularly when optimizing mix designs based on prediction outcomes, model interpretability becomes a critical requirement. SHAP, LIME, and other interpretability methods quantify the marginal contributions of features to prediction outcomes, enabling the revelation of model decision logic at both global and local levels. While significant progress has been achieved in applying these interpretability approaches in medical and financial domains [29,30,31], their implementation in civil engineering materials remains exploratory. Zhu et al. [32] integrated SHAP with an XGBoost model to analyze feature weights affecting the shear capacity of fiber-reinforced polymer–concrete interfaces, identifying fiber-reinforced polymer content as the most critical factor. Frie et al. [33] employed explainable machine learning to explore fatigue-influencing factors in materials. Yang et al. [34] developed ANN- and RF-based mortar design and optimization models, coupling them with interpretability analysis to reveal that cement content and apparent density of natural coarse aggregates exhibit the greatest impact on compressive strength. Jia et al. [35] leveraged SHAP to interpret LightGBM predictions, establishing an interpretable concrete mix proportion strength prediction model.

In the simulation of concrete mechanical behavior, numerical models widely employ discrete crack approaches and smeared damage approaches. Discrete crack approaches explicitly represent crack initiation and propagation, accurately capturing localization and softening behavior with high crack-path fidelity. For instance, De Maio et al. [36] proposed an adaptive cohesive interface model combined with a moving mesh technique to simulate arbitrary crack growth in heterogeneous materials. Smeared damage approaches (e.g., smeared cracks, continuum damage mechanics with internal variables) distribute cracking over the continuum; they are efficient for large-scale structural analyses but require regularization for mesh objectivity and rely on well-calibrated softening laws. Fan et al. [37] investigated the evolution of fatigue damage in concrete under uniaxial compression using sensors and X-ray computed tomography (CT) scans, providing an experimental basis for damage models.

Furthermore, hyperparameter optimization serves as a critical step for enhancing model performance, effectively improving generalization capabilities. Traditional grid search and random search methods suffer from inefficiency, while Bayesian optimization, genetic algorithms, and particle swarm optimization offer novel approaches for rapid tuning of complex models. Tran et al. [38] integrated Particle Swarm Optimization (PSO) with Extreme Gradient Boosting (XGBoost), Support Vector Regression (SVR), and Gradient Boosting (GB) to optimize hyperparameters for predicting the compressive strength of recycled concrete; this approach features simple parameter settings and fast convergence but is prone to local optima. To address this limitation, Zhang et al. [39] proposed an improved PSO where the inertia weight ω is dynamically adjusted with iterations to enhance particle swarm randomness and diversity, successfully applying it to mechanical parameter inversion analysis in dam engineering. Genetic algorithms (GA), grounded in principles of natural selection and genetic variation, enable global exploration of parameter spaces through crossover and mutation operations. Lim et al. [40] employed GA to optimize high-performance concrete mix design, while Ma et al. [41] developed four GA-machine learning models for predicting the shear performance of concrete beams, outperforming formula-based models in current international design codes. Beyond PSO and GA, Bayesian optimization exhibits unique advantages for hyperparameter tuning in concrete strength models: Zhang et al. [42] combined Gradient Boosted Decision Trees (GBDT) with Bayesian optimization to efficiently identify optimal hyperparameter combinations for recycled aggregate concrete tree models. Ragaa et al. [43] enhanced the generalization capability of neural networks predicting chloride and sulfate ingress into concrete using Bayesian optimization. Liu et al. [44] constructed precise concrete strength prediction models by leveraging Bayesian optimization for optimal algorithm selection and hyperparameter configuration.

Drawing from the existing literature, it is evident that while machine learning demonstrates significant potential for predicting concrete compressive strength, several critical gaps remain unaddressed. Firstly, although various ensemble algorithms have been applied, a systematic comparison of the latest Gradient Boosting Tree variants (XGBoost, LightGBM, CatBoost) against established methods like Random Forest and BPNN, using a controlled and consistent experimental dataset, is still lacking. This makes it difficult to identify the most robust algorithm for concrete mix-proportion problems. Secondly, the optimization process for these models often relies on traditional methods, leaving the full potential of efficient, advanced techniques like Bayesian optimization for this specific task underexplored. And the prevalent “black-box” nature of these high-performance models poses a major barrier to their practical adoption in engineering design.

**Figure 1:**
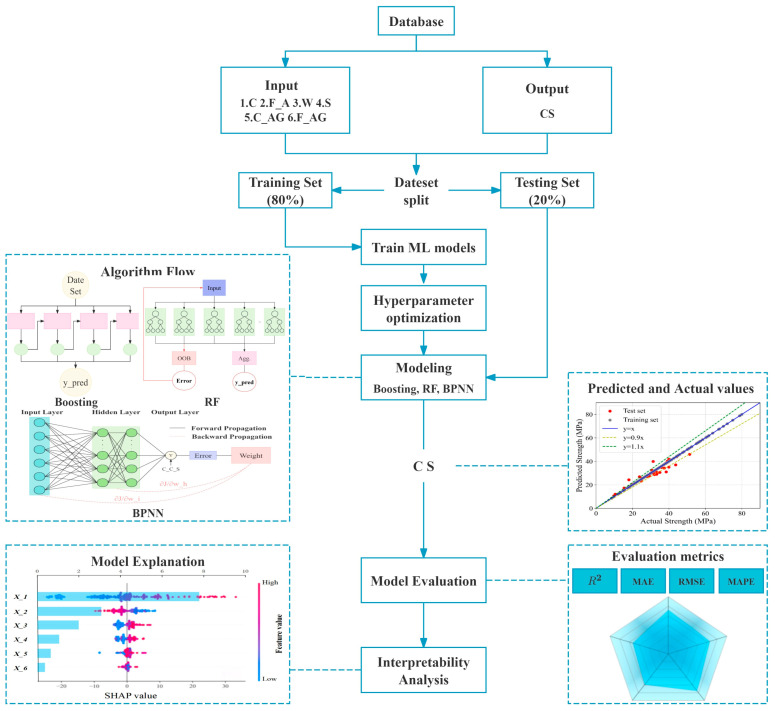
Flow Chart of Strength Prediction.

To address these issues, this study targets the prediction of 28-day compressive strength of concrete by adopting a machine learning framework integrating Bayesian optimization and interpretability analysis. Using 223 sets of mix proportion data obtained from field experiments, we systematically compare the predictive performance of five models: Gradient Boosting Trees (XGBoost, LightGBM, CatBoost), Random Forest (RF), and Backpropagation Neural Network (BPNN). Bayesian optimization is employed for efficient hyperparameter search to obtain optimal model parameters. Furthermore, SHAP methodology is introduced to quantitatively analyze the global contributions and local interaction effects of mix proportion parameters—including cement, fly ash, and water content—on compressive strength, revealing potential pathways for material proportion optimization (as illustrated in Figure 1). This research aims to overcome the dual challenges of precision in concrete mix design and model interpretability, providing an intelligent tool with both predictive power and decision transparency for engineering practice, ultimately driving the transformation of concrete material design from experience-driven to data-driven paradigms.

## 2. Research Method

### 2.1. Compression Test

**Figure 2:**
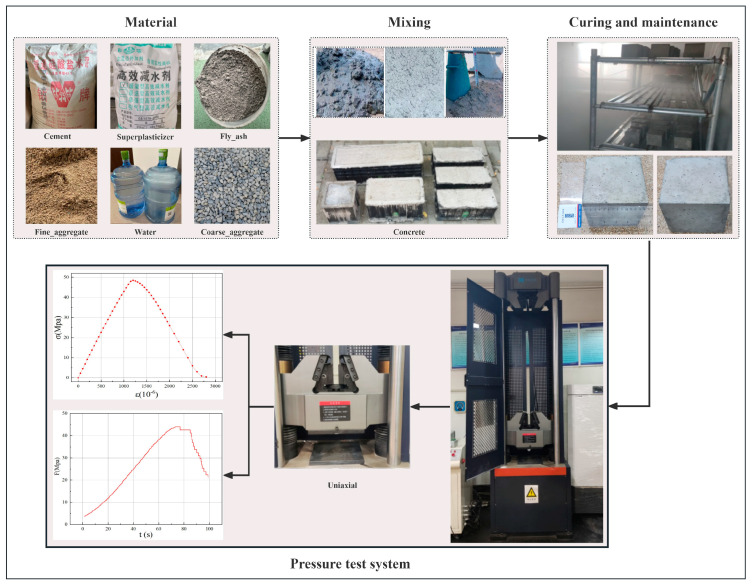
Specimen Preparation and Loading.

This study aims to develop a predictive model for concrete compressive strength, with its core focus on establishing the model’s input and output features. Concrete compressive strength is influenced by the coupled effects of multiple factors, and through literature review and engineering practice analysis, cement content, fly ash content, water–binder ratio, water reducer content, coarse aggregate gradation, and fine aggregate characteristics were selected as core input features. These parameters fundamentally determine the composition and internal structure of concrete mixtures, serving as primary determinants of its ultimate mechanical properties. According to current construction technical specifications, the mandatory core indicator for concrete structure quality acceptance is the cube compressive strength value after standard 28-day curing. This value must meet or exceed the design-specified strength grade. Therefore, this study explicitly selects the compressive strength value of concrete specimens under varying material mix proportions after standard 28-day curing as the output feature and target predictive variable of the model. To establish a high-quality sample dataset for model construction, concrete mix proportion compressive strength experiments were conducted at the central laboratory of a Beijing subway project, with the experimental procedure illustrated in Figure 2.

The experimental specimens were prepared in strict accordance with GB/T 50081-2019 Standard for Test Methods of Mechanical Properties of Ordinary Concrete Initially [45], six raw materials (cement, fly ash, water, water reducer, coarse aggregates, and fine aggregates) were precisely weighed based on a pre-designed experimental matrix covering diverse mix proportions; the materials were thoroughly homogenized using a mixer until the mixture achieved required workability, then cast into standard 150 mm × 150 mm × 150 mm cubic molds; subsequently, after full compaction on a vibrating table, specimens with molds underwent initial curing under controlled temperature (20 ± 2 °C) and humidity for 24 ± 2 h; following demolding, specimens were immediately transferred to a standard curing chamber maintaining 20 ± 2 °C temperature and ≥95% relative humidity for the designated 28-day period; upon completion of curing, specimens were extracted and their bearing surfaces precision-ground using a grinding machine to ensure planarity and full contact with loading plates; specimens were then subjected to uniaxial compressive loading via a hydraulic servo testing machine, which automatically recorded load–displacement curves (stress–strain) until failure occurred; the compressive strength of each specimen was precisely calculated based on the peak load at failure.

All experimental raw materials were rigorously selected with fixed fundamental parameters to control variables: cement comprised P·O 42.5-grade ordinary Portland cement; fly ash consisted of Class F Category II fly ash; fine aggregates employed qualified medium sand with moderate fineness modulus; coarse aggregates utilized well-graded crushed stone (5–25 mm particle size range); water reducer adopted retarding high-performance polycarboxylate superplasticizer; and mixing water used purified water meeting standard requirements. All materials were supplied by Yanxin Group, Beijing, China. All experimental batches strictly employed materials conforming to these specifications to ensure material performance consistency.

For each pre-designed mix proportion scheme, three fully independent replicate experiments were conducted (i.e., preparing three parallel specimens), with the arithmetic mean of test results from each set of three parallel specimens adopted as the representative value—this methodology effectively mitigates potential random errors in individual tests, enhancing data point stability and representativeness. Establishing a robust concrete compressive strength prediction model fundamentally requires sufficient high-quality sample data; to prevent model overfitting and increased prediction errors due to inadequate sample size, studies indicate that sample quantities typically need to be 20 to 50 times the number of features to provide adequate information for constraining model learning and capturing true feature-target relationships. This investigation planned and executed over 220 distinct mix proportion schemes, preparing and testing more than 660 concrete cube specimens; ultimately, through calculating the average strength of each set of three parallel specimens, 223 valid data points were obtained. These 223 structured data points constitute the core sample dataset for training and validating the concrete compressive strength prediction algorithm model in this study, satisfying fundamental data volume requirements for model training.

**Figure 3:**
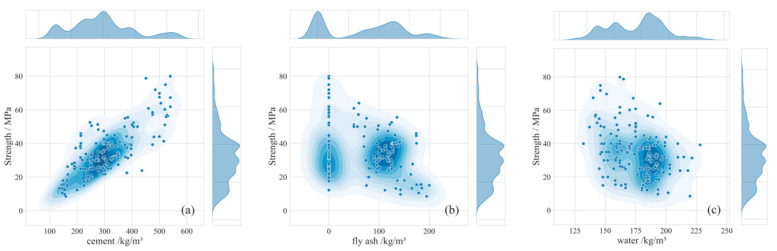

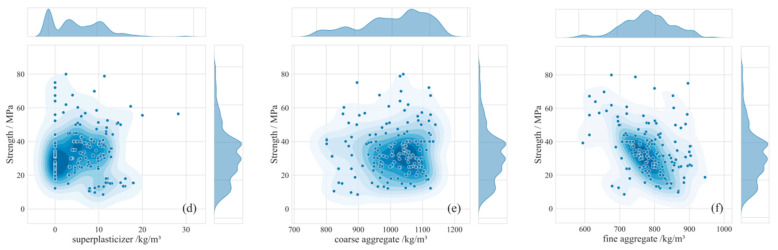
Distribution of Input Variables (**a**) cement; (**b**) fly ash; (**c**) water; (**d**) superplasticizer; (**e**) coarse aggregate; (**f**) fine aggregate.

Figure 3 intuitively visualizes the univariate relationships between each of the six input features and concrete compressive strength through scatter plots, revealing key distribution characteristics of the dataset; the distribution patterns of scatter points in each subplot clearly illustrate the overall impact direction of changes in the dosage of individual material components on compressive strength. Specifically, cement dosage (ranging from 100 kg/m^3^ to 600 kg/m^3^) generally shows a positive correlation with strength, while water content (ranging from 125 kg/m^3^ to 230 kg/m^3^) predominantly exhibits a significant negative correlation.

By observing the distribution histograms of each feature, the data distributions exhibit approximately symmetrical bell-shaped curves consistent with fundamental characteristics of normal distributions. While some features may display right-skewed distributions: data clusters in lower-value regions and tails off toward higher values, a pattern characteristic of log-normal distributions. The overall normal or near-normal distribution characteristics of the data hold significant statistical importance, establishing a valid prerequisite for subsequent quantitative examination of linear correlations between features and strength using Pearson’s correlation coefficient.

### 2.2. Data Pre-Processing

#### 2.2.1. Correlation Analysis

To eliminate redundant features among variables, effectively reduce data dimensionality, enhance training efficiency, and mitigate overfitting risks, correlation analysis of the data must be conducted prior to machine learning [46]. Correlation analysis is a statistical methodology for investigating the degree and nature of associations between two or more variables. Primary correlation metrics include the Pearson correlation coefficient, Spearman’s rank correlation coefficient, and Kendall’s rank correlation coefficient. Among these, the Pearson correlation coefficient is the most widely used method for detecting linear relationships, requiring continuous variables with jointly normally distributed data. This study employs the Pearson correlation coefficient for feature correlation assessment, computed as follows (Equation (1)):

(1) $$r_{XY}=\frac{\sum_{i=1}^{n}(x_{i}-\bar{x})(y_{i}-\bar{y})}{\sqrt{\sum_{i=1}^{n}{\left(x_{i}-\bar{x}\right)}^{2}\sum_{i=1}^{n}{\left(y_{i}-\bar{y}\right)}^{2}}}$$

where $x_{i}$ and $y_{i}$ denote sample observations, $\bar{x}$ and $\bar{y}$ represent sample means, and $r_{XY}$ ranges within [−1, 1] Values approaching 1 or −1 indicate strong linear correlation, while values near 0 signify weak linear correlation.

**Figure 4:**
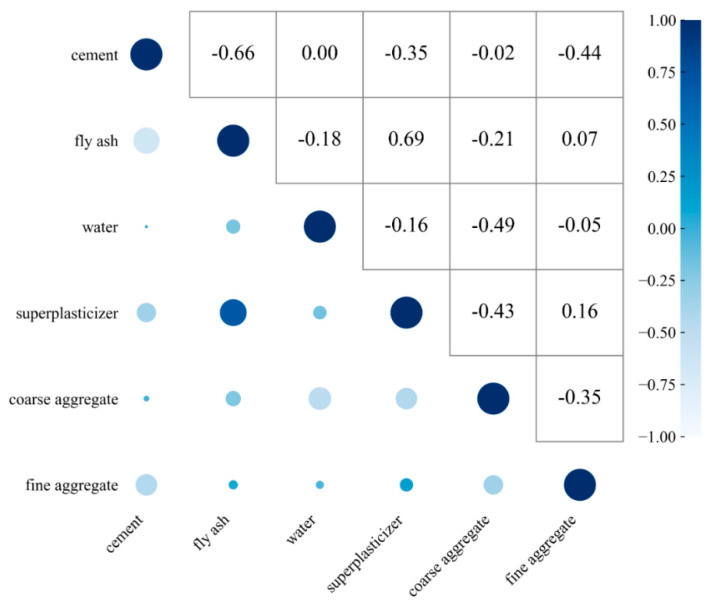
Correlation Heatmap of Concrete Mix Proportion Parameters.

Figure 4 presents a heatmap illustrating the correlations among six concrete mix proportion parameters. This visualization employs a color-mapping matrix where dark hues (approaching positive values) indicate strong positive correlations and light hues (approaching negative values) denote strong negative correlations. For instance, the correlation coefficient between fly ash and fine aggregates is 0.69, numerically and visually demonstrating a strong positive relationship; conversely, cement and fly ash exhibit a strong negative correlation (−0.66). Machine learning implementation is generally considered feasible when inter-feature correlation coefficients remain below 0.8. The heatmap in Figure 4 confirms that all correlation magnitudes, whether positive or negative, fall within acceptable thresholds, thereby confirming the absence of multicollinearity issues in the dataset.

#### 2.2.2. Data Normalization

Data normalization plays a critical role in machine learning by standardizing feature scales to accelerate convergence of optimization algorithms (e.g., gradient descent), reduce training time, and mitigate bias caused by magnitude discrepancies across features. This process enables balanced feature weighting learning, enhances prediction accuracy, and improves generalization capability. The specific normalization method is computed as follows (Equation (2)), mapping results to the [0, 1] interval.

(2) $$X_{scaled}=\frac{X-X_{min}}{X_{max}-X_{min}}$$

where *X* denotes the original value of a feature; *X_min_* denotes the minimum value of that feature observed across the entire dataset; *X_max_* denotes the maximum value of that feature observed across the entire dataset and *X_scaled_* denotes the resulting normalized value in the [0, 1] range.

**Figure 5:**
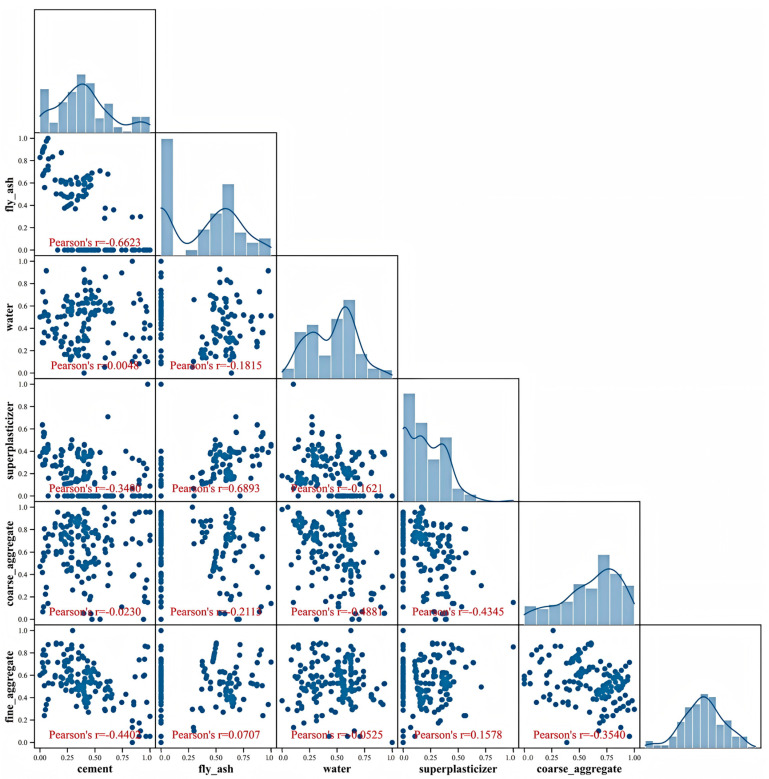
Scatter Plot Matrix with Marginal Histograms of Parameters.

Utilizing normalized data, a matrix scatter plot with marginal histograms was generated to visualize the distribution patterns of concrete mix proportion parameters. Figure 5 presents this matrix visualization of scatter plots and edge histograms for concrete mix parameters. Along the main diagonal, normalized value histograms for each parameter display their probability distribution morphology, while off-diagonal plots illustrate scatter relationships between any two parameters, revealing feature correlations. The histograms distinctly demonstrate that most parameters (e.g., cement content, aggregate quantities) exhibit distributions highly consistent with normal distributions, whereas skewed features (e.g., water reducer dosage) conform to log-normal distribution characteristics. This distribution behavior aligns with the analysis in Section 2.1, further validating the appropriateness of employing Pearson correlation coefficients for linear correlation analysis. The detailed statistics of characteristic parameters are shown in Table 1.

### 2.3. Compressive Strength Prediction Model Construction

**Figure 6:**
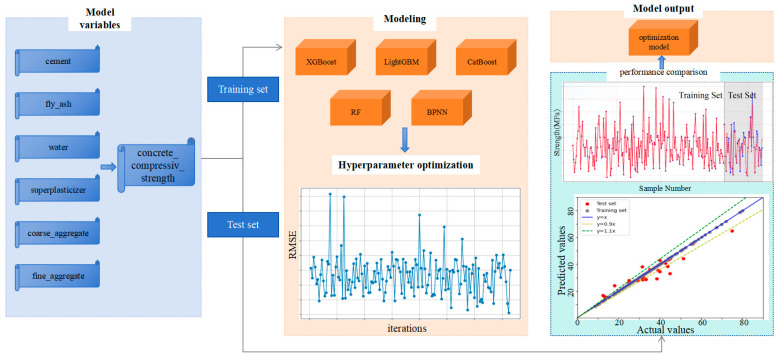
Model Building Process.

In this study, Gradient Boosting Tree algorithms (XGBoost, LightGBM, CatBoost), Random Forest (RF), and Backpropagation Neural Network (BPNN) were adopted to establish concrete strength prediction models. The dataset was initially partitioned into training and test sets at an 8:2 ratio, with the training set used for model learning and parameter optimization, and the independent test set reserved for evaluating generalization performance. The modeling framework comprised three core phases: (1) selection and preprocessing of datasets, (2) determination of modeling algorithms combined with hyperparameter optimization, and (3) evaluation of models using regression metrics, including the coefficient of determination (R^2^), mean squared error (MSE), and root mean squared error (RMSE) (As shown in Figure 6). To quantify uncertainty in model performance, bootstrap resampling with 1000 iterations was applied to the test set, from which median values and 95% confidence intervals of R^2^, RMSE, MAE, and MAPE were derived, providing robust estimates of performance variability. Comparative metric analysis was then used to identify the optimal concrete strength prediction model.

### 2.4. Hyperparameter Optimization

Machine learning hyperparameters are parameters that must be manually set prior to model training, with model performance critically dependent on their selection; these hyperparameters govern model complexity and influence both the training process and generalization capability, necessitating optimization before model training. Taking the n_estimators parameter in the XGBoost model as an example, the hyperparameter optimization process is introduced below.

In machine learning, the metric used to evaluate model accuracy on unseen data is termed the generalization error. For an ensemble model *f*, its generalization error *E(f:D)* on an unknown dataset *D* (as expressed in Equation (3)) is determined by the combined effects of variance (*var*), bias (*bias*), and noise (*ε*). bias reflects the degree of fit on the training data, var indicates model stability, and *ε* represents inherently uncontrollable disturbances. A smaller generalization error corresponds to a more ideal model.

(3) $$E(f:D)=bias^{2}+var+\varepsilon^{2}$$

**Figure 7:**
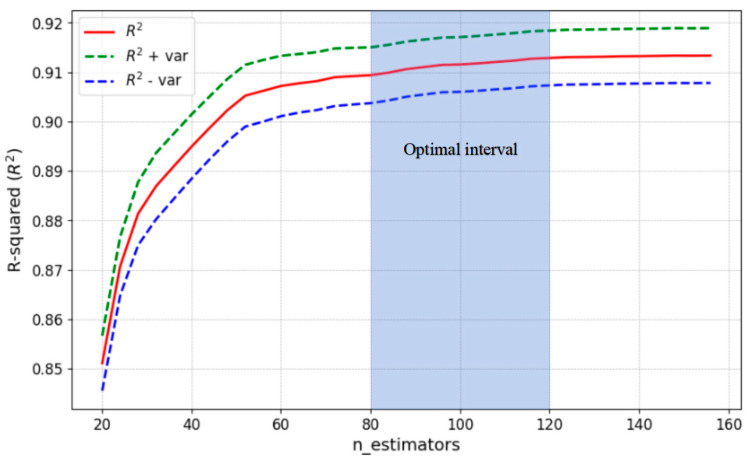
Learning curve.

Optimizing solely for the highest score on the learning curve (i.e., the point of minimal bias) introduces substantial hyperparameter tuning error due to neglected variance, thus, the optimization process must holistically incorporate variance. Figure 7 illustrates the model’s fitting performance (R^2^ score) across varying *n_estimators* values and its generalization capability assessment. The solid line depicts the trend of R^2^ scores versus *n_estimators*, while the green dashed line and blue dashed line represent R^2^ ± the variance from 5-fold cross-validation at each point—quantifying model stability (closer proximity of dashed lines to the solid line indicates lower variance and greater stability). Key observations from Figure 7 reveal: as *n_estimators* increases, R^2^ scores rise continuously, plateauing beyond 80. R^2^-focused optimization alone would suggest setting *n_estimators* between 80–120. However, variance analysis demonstrates that at low *n_estimators* values, the upper/lower variance boundaries converge toward the solid line; with increasing *n_estimators* (elevated model complexity), *bias* decreases (higher R^2^) but variance gradually amplifies, stabilizing after *n_estimators* exceeds 60. Consequently, balancing R^2^ (fitting performance) and variance (generalization stability) warrants initializing *n_estimators* within 80–120.

**Figure 8:**
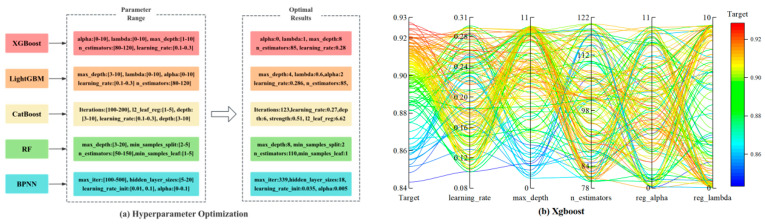

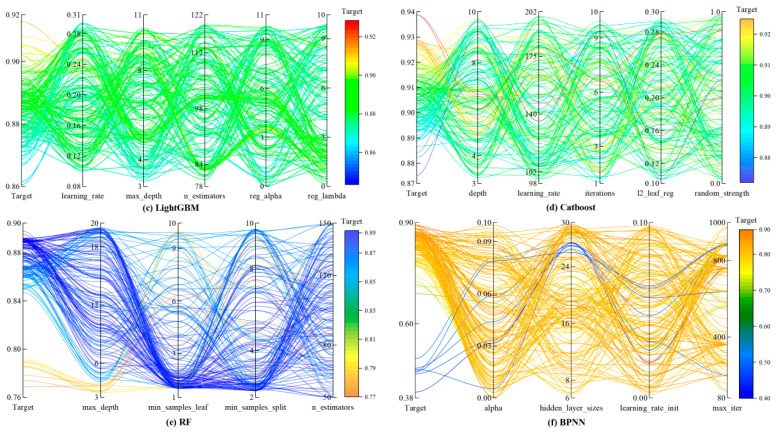
Bayesian Algorithm Hyperparameter Optimization.

The hyperparameter optimization process for other parameters follows a similar methodology. Initially, delineate search ranges is achieved by plotting learning curves for key hyperparameters to observe performance trends on both training and validation sets. Subsequently, within this preliminary parameter space, advanced hyperparameter optimization algorithms automatically seek optimal configurations. For example, Bayesian optimization efficiently leverages existing data to identify model optima with minimal iterations. Figure 8 presents the defined hyperparameter search ranges for different models alongside Bayesian optimization results.

### 2.5. Model Evaluation Methodology

Model evaluation employs a multi-dimensional indicator system, integrating traditional performance metrics with cross-validation strategies to ensure the reliability and generalizability capability. This study employs the Coefficient of Determination (*R^2^*), Mean Absolute Error (*MAE*), Root Mean Square Error (*RMSE*), and Mean Absolute Percentage Error (*MAPE*) to comprehensively evaluate the predictive performance of the model [47,48].

The coefficient of determination (*R*^2^) measures the proportion of variance in the dependent variable explained by the regression model, with values ranging from 0 to 1. Its calculation is given in Equation (4).

(4) $$R^{2}=1-\frac{\sum_{i=1}^{n}{\left(y_{i}-\hat{y_{i}}\right)}^{2}}{\sum_{i=1}^{n}{\left(y_{i}-\bar{y_{i}}\right)}^{2}}$$

The Mean Absolute Error (*MAE*) is the arithmetic mean of the absolute differences between predicted values and actual values. It is less sensitive to outliers and reflects the average level of error. Its calculation can be performed using Equation (5).

(5) $$MAE=\frac{1}{n}\sum_{i=1}^{n}\left|y_{i}-\hat{y_{i}}\right|$$

The Root Mean Square Error (*RMSE*) is the square root of the average of the squared differences between predicted values and actual values. Due to the squaring operation amplifying the effect of larger errors, it is more sensitive to outliers and emphasizes the dispersion of the errors. Its calculation can be performed using Equation (6).

(6) $$RMSE=\sqrt{\frac{1}{n}\sum_{i=1}^{n}{\left(y_{i}-\hat{y_{i}}\right)}^{2}}$$

The Mean Absolute Percentage Error (*MAPE*) is the arithmetic mean of the absolute deviation between the predicted value and the actual value, expressed as a percentage of the actual value. As a relative measure of error, it is suitable for comparing errors across datasets with different scales. Its calculation is presented in Equation (7).

(7) $$MAPE=\frac{1}{n}\sum_{i=1}^{n}\left|\frac{y_{i}-\hat{y_{i}}}{y_{i}}\right|\times 100\%$$

### 2.6. Interpretability Analysis

SHAP (SHapley Additive exPlanations), grounded in Shapley values from cooperative game theory, quantifies feature contributions to prediction outcomes (including magnitude and directionality) [49]. The additivity property of SHAP enables clear decomposition of each feature’s contribution, where the predicted value can be expressed as the sum of all feature SHAP values and a baseline prediction (Equation (8)).

(8) $$\hat{y}(x)=y_{base}+\sum_{i=1}^{n}\phi_{i}(x)$$

where $\phi_{i}(x)$ denotes the SHAP value of feature *i* for sample *x*, and *y_base_* represents the baseline prediction.

### 2.7. Computational Implementation and Reproducibility

To ensure the reproducibility of our experiments, all models were developed using Python 3.13. The primary machine learning libraries employed were scikit-learn (version 1.6.1) for data preprocessing, Random Forest, and train-test splitting; XGBoost (version 3.0.0), LightGBM (version 4.6.0), and CatBoost (version 1.2.8) for the gradient boosting models; and SHAP (version 0.48.0) for interpretability analysis. The Bayesian optimization was implemented using the BayesSearchCV class from the scikit-optimize (version 0.6.6) library. All experiments were performed on a computer with an Intel Core i7-13790F CPU, 16 GB RAM, and an NVIDIA GeForce RTX 4060 Ti GPU. We expect to be able to easily process and implement the research framework in this paper with an ordinary personal computer.

## 3. Results and Analysis

### 3.1. Comparison and Evaluation of Algorithm Model

**Figure 9:**
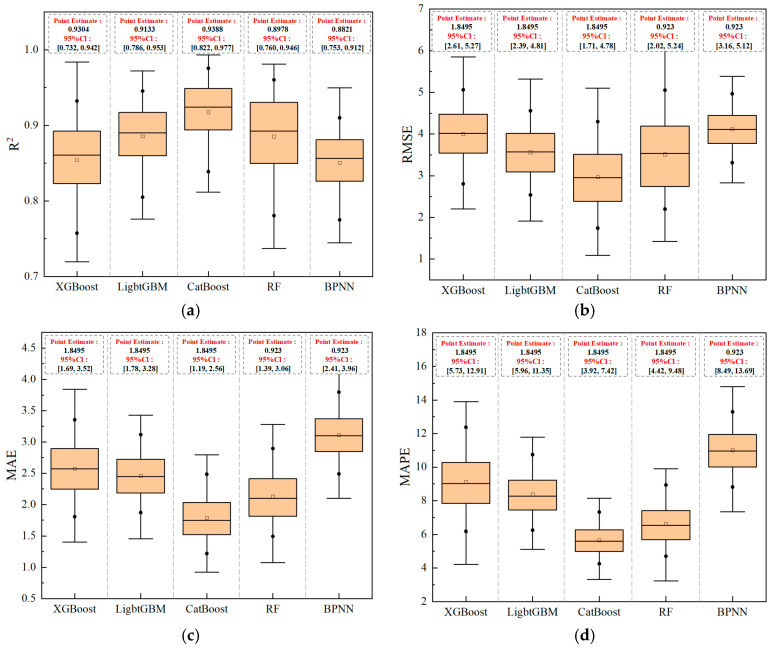
Box Plots of Performance Metrics for Different Machine Learning Model (**a**) R^2^, (**b**) RMSE, (**c**) MAE, (**d**) MAPE.

All five machine learning models discussed in this section were trained using the optimal hyperparameters identified in Section 2.4. Figure 9 presents box plots of the four core evaluation metrics, while Table 2 provides point estimates of these metrics along with individual metric scores and comprehensive rankings. This approach not only reveals the stability of model performance across repeated validations but also quantifies the absolute performance of each model.

As an effective statistical visualization tool, the box plots illustrate the distribution characteristics of each metric. In Figure 9, the boxes represent the interquartile range (IQR), the horizontal line inside each box indicates the median, the small black square denotes the mean. Overall, CatBoost demonstrates superior performance across all metrics: its box is positioned highest in the R^2^ subplot (reflecting the best data fitting ability, with a median of 0.924), and lowest in the RMSE, MAE, and MAPE subplots (indicating the smallest prediction errors, with median values of 2.97 MPa, 1.77 MPa, and 5.64%, respectively). Moreover, CatBoost exhibits the narrowest 95% confidence intervals among all models (R^2^: [0.822, 0.977], RMSE: [1.707, 4.477], MAE: [1.190, 2.564], MAPE: [3.924, 7.419]), confirming its exceptional performance stability.

Table 2 summarizes the evaluation metrics for the five machine learning models. The values presented are point estimates calculated on the original full test set, not bootstrap medians. The gradient boosting tree models—CatBoost, XGBoost, and LightGBM—collectively achieved R^2^ values above 0.9, indicating strong data fitting performance. In contrast, Random Forest (RF) and the Backpropagation Neural Network (BPNN) yielded R^2^ values below 0.9, reflecting relatively weaker fitting efficacy. Specifically, CatBoost attained the highest R^2^ (0.9388), outperforming XGBoost (0.9304) and LightGBM (0.9133). In terms of error metrics, CatBoost also demonstrated lower RMSE (2.7131 MPa) and MAPE (5.45%) than the other two models. Although its MAE (1.9786 MPa) was slightly higher than that of XGBoost (1.8495 MPa), the difference was negligible. Therefore, CatBoost exhibits the best overall performance in terms of goodness-of-fit and error control, confirming its strong capability to accurately predict compressive strength across diverse mix proportions.

**Figure 10:**
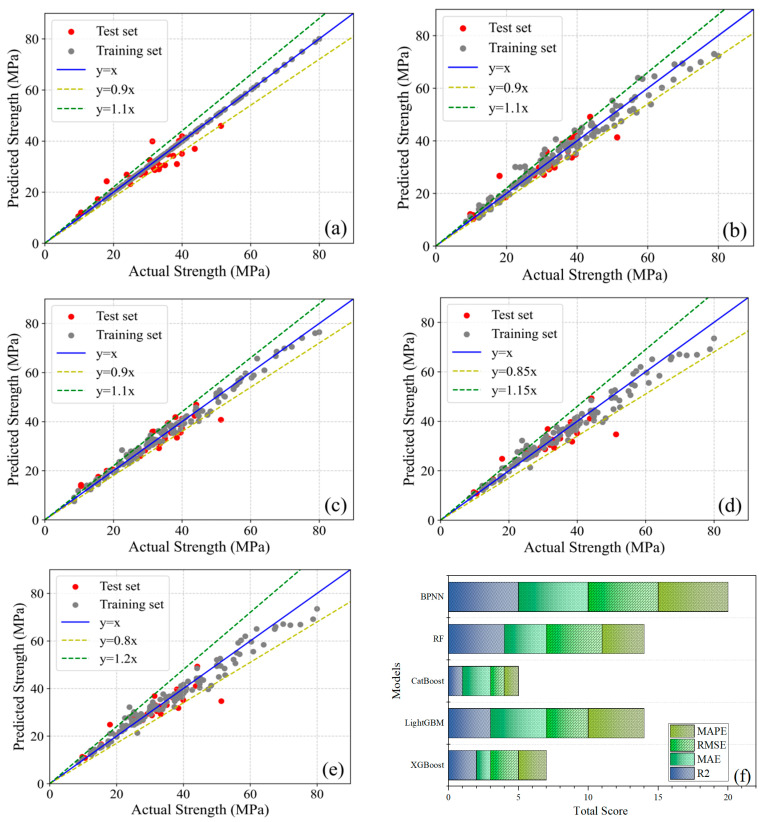
Comparison of Actual vs. Predicted Compressive Strength (**a**) XGBoost; (**b**) LightGBM; (**c**) CatBoost; (**d**) RF; (**e**) BPNN; (**f**) R^2^ Score.

Figure 10 compares predicted versus actual concrete compressive strengths across five machine learning models using scatter plots with reference lines, where red and grey markers denote test and training sets, respectively; the blue line (y = x) represents the ideal prediction state, while yellow and green lines demarcate specific deviation ranges. Training set fit reflects prediction accuracy, whereas test set fit indicates generalization capability—analysis reveals minor deviations between predicted and actual values for most data points across all models, confirming robust predictive performance primarily attributable to Bayesian optimization tuning. Notably, Gradient Boosting Tree models (CatBoost, XGBoost, LightGBM) exhibit > 95% of data points within ±10% error bounds, whereas Random Forest (RF) and Backpropagation Neural Network (BPNN) demonstrate 15–20% fitting errors, establishing GBDT superiority. Critically, XGBoost’s near-perfect training alignment contrasts with its test R^2^ (0.9304), indicating overfitting and elevated generalization error. In contrast, CatBoost maintains uniform distribution of both training and test points within 10% error bounds, achieving superior test R^2^ (0.9388) with minimal overfitting and optimized generalization.

**Figure 11:**
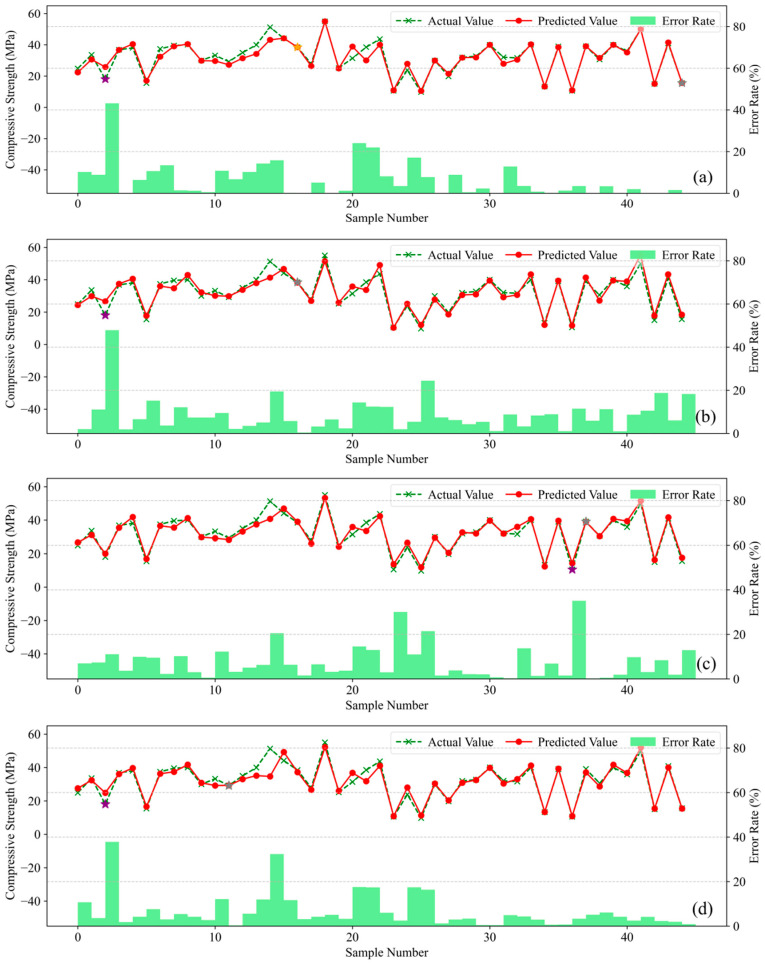

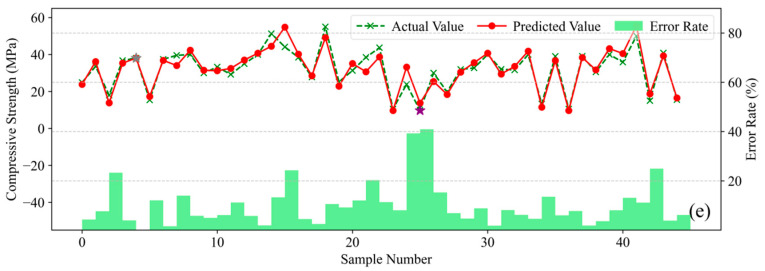
Error Representation: (**a**) XGBoost; (**b**) LightGBM; (**c**) CatBoost; (**d**) RF; (**e**) BPNN.

Figure 11 presents the prediction error analysis for the five machine learning models. The green polyline represents actual compressive strength values, the red polyline denotes model-predicted values, and green bars indicate the relative prediction error for each sample; comparison between the green and red polylines visually reveals prediction deviations per sample, while the error bars quantitatively illustrate the magnitude of divergence between predicted and actual values.

Analysis reveals that Gradient Boosting Tree models achieve close alignment between predicted and actual values on the training set. With only isolated exceptions exceeding 20% error, CatBoost, XGBoost, and LightGBM maintain relative errors within 10% for most samples. These models exhibit Mean Absolute Percentage Errors (MAPE) of 5.45%, 6.44%, and 8.71%, respectively. In contrast, Random Forest (RF) and Backpropagation Neural Network (BPNN) yield higher MAPEs of 6.71% and 10.13%. Collectively, these results confirm CatBoost’s superior accuracy in predicting concrete compressive strength across diverse mix proportions.

Consequently, among the five evaluated models, CatBoost demonstrates optimal comprehensive performance with superior compatibility for this concrete mix proportion strength dataset. While XGBoost exhibits marginally inferior performance to CatBoost yet maintains competent predictive capability, whereas Random Forest (RF) and Backpropagation Neural Network (BPNN) deliver comparatively weaker overall performance.

### 3.2. Comparison and Evaluation of Empirical Formulas

To objectively assess the engineering applicability of the optimized CatBoost model, this study systematically compares its predictive performance with the current industry standard Specification for Mix Proportion Design of Ordinary Concrete (JGJ 55-2011) [50]. According to JGJ 55, Equation (9) is used to calculate and predict the strength of concrete.

(9) $$f_{cu,pred}=\alpha_{a}\cdot f_{ce}\cdot (\frac{C+FA}{W}-\alpha_{b})$$

where *f_cu,pred_* denotes the predicted cube compressive strength (MPa); *α_a_* and *α_b_* are regression coefficients (0.53 and 0.20, respectively); *f_c__e_* is the actual compressive strength of cement (MPa); *C* represents cement content (kg/m^3^); *FA* indicates mineral admixture content (kg/m^3^); and *W* is water content (kg/m^3^).

Ten sets of validation experiments were designed, with concrete mix proportions detailed in Table 3.

**Figure 12:**
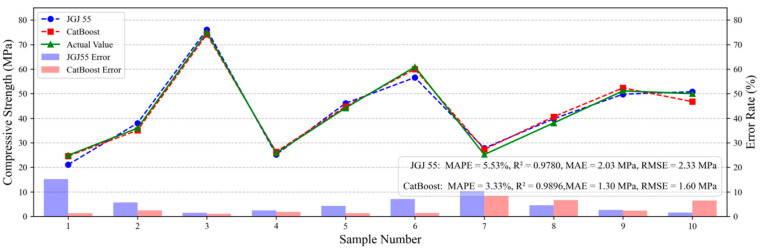
Performance Evaluation and Error Comparison.

The ten sets of mix proportion parameters were input into Equation (9) and the established CatBoost prediction model, with results presented in Figure 12. The red curve represents strength values predicted by the CatBoost model, the blue curve denotes calculated values from the empirical formula in the Design Code (Equation (9)), and the green curve indicates experimentally measured actual strength values. An accompanying bar chart displays error rates between the Design Code predictions, CatBoost predictions, and actual strength measurements.

Both empirical formula (Equation (9)) and the CatBoost model demonstrate competent concrete strength prediction, yet CatBoost achieves a lower mean absolute percentage error (MAPE = 2.94%) compared to the empirical formula (MAPE = 5.96%). Superiority of the CatBoost model is further evidenced by metrics including R^2^ score, MAE, and RMSE. Further analysis reveals that the core strength calculation in JGJ 55-2011 does not explicitly incorporate key parameters, such as water reducer dosage, coarse/fine aggregate quantities, or critical aggregate properties as direct variables. Instead, their influences are indirectly addressed through: (1) water reducer’s impact via water reduction rate (affecting water content), (2) aggregate effects via regression coefficients and sand ratio optimization, (3) Mandatory trial batching to derive qualified and economical mix proportions. The CatBoost model overcomes these simplifications by accurately capturing nonlinear couplings among admixtures, additives, and material properties. Thus, under the specific experimental conditions (P·O 42.5 cement, Class F Category II fly ash, graded aggregates, and retarding high-performance polycarboxylate superplasticizer), the CatBoost model marginally outperforms the empirical formula from Specification for Mix Proportion Design of Ordinary Concrete (JGJ 55-2011) in single-point strength prediction accuracy.

### 3.3. External Validation on UCI Dataset

To further evaluate the generalization performance of the proposed model, we conducted external validation on the Concrete Compressive Strength dataset from the UCI Machine Learning Repository. This dataset contains a total of 1030 samples, each with 8 input features (including cement, blast furnace slag, fly ash, water, superplasticizer, coarse aggregate, fine aggregate, and age) and the corresponding compressive strength value. It should be noted that the experimental dataset used in this paper only contains six features (cement, fly ash, water, superplasticizer, coarse aggregate, fine aggregate), indicating a feature discrepancy with the UCI dataset. To address this discrepancy, we adopted two evaluation strategies: Strategy A selected samples from the UCI dataset with a blast furnace slag content of 0 and an age of 28 days, obtaining a total of 175 samples. Strategy B selected 311 samples with a blast furnace slag content of 0 and an age not less than 28 days. The age of 28 days was chosen as the key age because concrete strength typically reaches over 90% of its final strength at this stage, and subsequent strength gain tends to stabilize, making age no longer a primary influencing factor. Furthermore, the larger sample size in Strategy B helps to more comprehensively test the model’s generalization capability.

The trained CatBoost model was directly applied to the aforementioned two UCI subsets without any retraining or parameter fine-tuning. The prediction performance results are summarized in Table 4.

On the UCI subset with a higher degree of feature matching (Strategy A), the CatBoost model achieved a prediction performance of R^2^ = 0.9014, RMSE = 3.5827, and MAPE = 11.21%. Although slightly lower than the performance on the internal test set, potentially due to differences in material sources, curing conditions, and experimental procedures, the overall metrics remain at a high and acceptable level, indicating that the model maintains good predictive capability even outside the distribution of the training data.

For the Strategy B subset, which has a broader sample range but suffers from age feature mismatch, the model performance showed a further expected decline (R^2^ = 0.7480, RMSE = 6.9738, MAPE = 12.15%). This reflects the challenges faced when the key variable “Age” is missing from the model’s input features. It also indicates that the model’s prediction accuracy is affected when there are significant distributional differences between the external data and the training data. Nonetheless, the model maintained considerable predictive effectiveness in terms of R^2^ and error metrics and did not completely fail, further confirming its certain degree of robustness.

In summary, the external validation results on the UCI dataset demonstrate that the CatBoost model developed in this study possesses good generalization capability. Particularly on external data with good feature matching (Strategy A), the model can provide high-accuracy predictions comparable to those on the internal test set. Even in more challenging scenarios with incomplete feature matching and greater data distribution differences (Strategy B), the model can still output reference-worthy prediction results.

### 3.4. SHAP Interpretability Framework for Feature Importance Analysis

#### 3.4.1. Global Interpretation and Analysis

The inherent opacity of machine learning models impedes explanatory insight into predictive outcomes, thereby compromising model trustworthiness. To resolve this limitation, we integrate the optimized CatBoost architecture with SHAP (SHapley Additive exPlanations) interpretability framework. This synthesis quantifies interaction mechanisms between concrete mix proportion parameters (inputs) and compressive strength (output), establishing a systematic quantification paradigm for evaluating compositional influences on mechanical performance.

**Figure 13:**
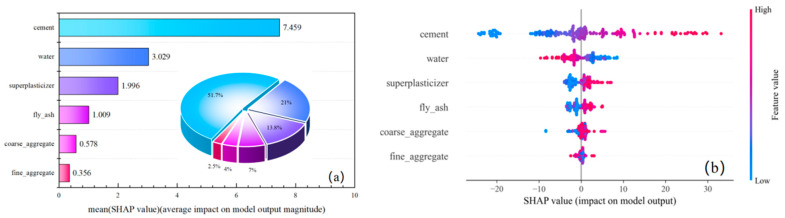
SHAP Value Summary for Input Features (CatBoost). (**a**) Relative importance of features; (**b**) Shapley Value Contribution.

Figure 13 presents the SHAP summary plot for input features of the optimal CatBoost model, where the left panel displays global feature importance represented by mean absolute SHAP values across all samples. Cement exhibits the highest mean SHAP value (7.459), confirming its dominant influence on compressive strength predictions and establishing it as the most critical factor. The features are ranked in descending order of impact magnitude: cement > water > superplasticizer > fly ash > coarse aggregate > fine aggregate. The right panel visualizes feature effects through SHAP distributions. The horizontal axis denotes SHAP magnitude (negative values reduce output strength while positive values enhance it, with absolute magnitude indicating effect intensity); vertical ordering reflects influence hierarchy; color mapping indicates feature values (blue for low values, red for high values). Cement demonstrates complex dichotomous behavior: low values (blue) predominantly exert negative effects while high values (red) enhance strength. Superplasticizer, fly ash, and both aggregates exhibit consistent positive relationships with strength. Water displays threshold-dependent effects: lower content potentially increases strength whereas higher content diminishes it.

#### 3.4.2. Feature Interaction Interpretation

**Figure 14:**
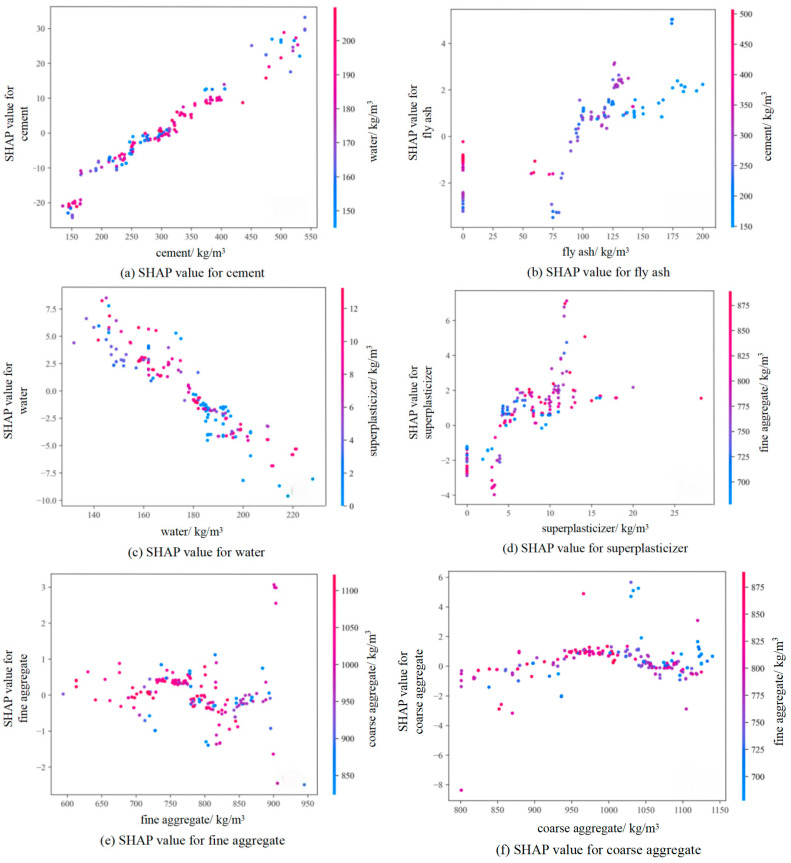
SHAP Dependence Plot for Features.

Figure 14 presents SHAP dependence plots to visualize individual feature impacts on model outputs and their interactive effects; as shown in Figure 14a, SHAP values exhibit significant fluctuations (−20 to +20) within the 200–500 kg/m^3^ cement dosage range, yet demonstrate an overall positive correlation trend. Increasing cement dosage consistently elevates SHAP values, confirming cement’s substantial positive contribution to compressive strength predictions, with higher dosages being particularly critical for strength enhancement. Color gradients represent water content, revealing that under high-water conditions, cement increases correspond to more pronounced SHAP value elevations. This evidence indicates water content modulates cement’s strengthening efficacy, demonstrating a synergistic interaction effect where cement’s strength-enhancing potential is amplified under elevated water conditions.

As fly ash content increases, SHAP values initially decrease, then rise, and subsequently fluctuate—with more pronounced variation amplitudes occurring under high-cement conditions. This signifies a synergistic interaction between cement content and fly ash’s impact on concrete strength, where fly ash variations exert greater influence on strength development at high cement levels, as demonstrated in Figure 14b. Concurrently, increasing fly ash correlates with decreasing cement content, leading to reduced SHAP values; this substitution relationship indicates that excessive cement replacement by fly ash may compromise concrete performance.

In the SHAP dependence plot for water, Figure 14c, SHAP values decrease significantly from +10 to −8 as water content increases (150–200 kg/m^3^), demonstrating a strong negative correlation that confirms excessive water reduces compressive strength; however, this negative effect is partially mitigated under high superplasticizer content conditions, indicating that superplasticizers improve strength by reducing water demand, whereby a notable synergistic interaction exists between these parameters.

Coarse and fine aggregate contents exhibit no discernible monotonic trends with SHAP values, as shown in Figure 14e,f, both demonstrate optimal content ranges where they contribute favorably to concrete strength, beyond which their effects become variable and less predictable.

#### 3.4.3. Local Explanation and Analysis

**Figure 15:**
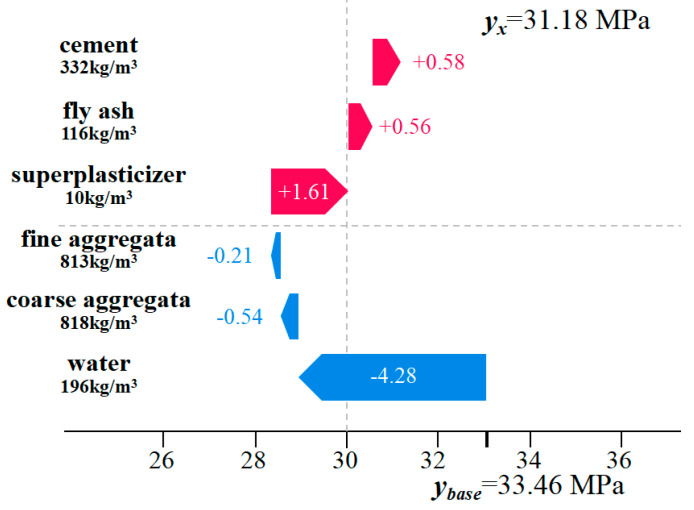
SHAP Force Plot for Compressive Strength (Local Explanation).

Local explanation is another interpretative approach provided by the SHAP method, used to interpret the prediction outcomes for individual samples within a dataset. For both global and local explanations, the sign (positive/negative) of the SHAP value represents the magnitude and direction of the driving force. The model prediction value equals the sum of the feature SHAP values and the baseline value. Figure 15 illustrates the explanatory force plot for a sample randomly selected from the input dataset. In the figure, the baseline value for concrete strength is 33.46 MPa, while the actual predicted value is 31.18 MPa. The red arrow pointing left represents the direction of higher values, while the blue arrow pointing right represents the direction of lower values. Cement, fly ash, and superplasticizer are positioned in the direction of the red arrow, indicating that an increase in these component values would shift the model output above the baseline value of 31.18 MPa. Cement exhibits the longest force arm, signifying its substantial positive contribution to compressive strength. Conversely, water, coarse aggregate, and fine aggregate are positioned in the direction of the blue arrow, indicating that an increase in these component values would shift the model output below the baseline value.

## 4. Conclusions

This study systematically established a predictive model for the 28-day compressive strength of concrete based on an interpretable machine learning framework, integrating Bayesian optimization with the SHAP method. This research further investigated the key influencing mechanisms of material mix proportions on strength. Through modeling and optimization analysis of 223 sets of experimental data, the following main conclusions are drawn:

(1) Among the five predictive models, namely Gradient Boosting Trees, the CatBoost model, after Bayesian Optimization of its hyperparameters, demonstrated the best performance. Its coefficient of determination (R^2^) reached 0.9388, root mean square error (RMSE) was 2.7131 MPa, and mean absolute percentage error (MAPE) was 5.45%. All performance metrics were significantly superior to those of the other comparative models. Furthermore, box plot analysis from repeated validations confirmed that CatBoost exhibits the narrowest 95% confidence intervals (R^2^: [0.822, 0.977], RMSE: [1.707, 4.477], MAPE: [3.924, 7.419]), indicating superior performance stability.

(2) The integration of the machine learning framework with the Bayesian optimization algorithm plays a critical role in enhancing model predictive performance. Following hyperparameter optimization, not only was model accuracy improved, but the model’s generalization capability was also strengthened, validating the applicability of this approach to optimizing complex machine learning models.

(3) Compared with the empirical formulas specified in the current industry standard Specification for Mix Proportion Design of Ordinary Concrete (JGJ 55-2011), the CatBoost model established in this study demonstrates superior single-strength prediction accuracy under specific raw materials (e.g., P·O 42.5 cement, Class F Grade II fly ash) and curing conditions, achieving a mean absolute percentage error (MAPE) of 2.94%. This validates the advantage of data-driven models in capturing complex nonlinear interactions among materials. External validation on the UCI dataset confirmed the model’s strong generalization capability and practical applicability in cross-dataset scenarios, achieving an R^2^ of 0.9014 on a well-matched feature subset and maintaining reasonable predictive performance (R^2^ = 0.7480) even under incomplete feature matching.

(4) SHAP analysis reveals that cement content is the most influential feature affecting concrete strength (mean SHAP value: 7.459), followed by water, superplasticizer, fly ash, and coarse/fine aggregates. Global interpretation uncovers nonlinear relationships among features: a synergistic effect between high cement content and appropriate superplasticizer dosage enhances strength, while excessive water significantly compromises compressive strength. Local dependence analysis further demonstrates that the cement–water interaction is particularly pronounced under high water–cement ratios, and the substitution effect of fly ash requires dynamic optimization adjustments based on cement content.

It is important to note that this study was conducted under standardized laboratory curing conditions (20 °C, ≥95% RH), which, while enabling a clear interpretation of mix proportion effects, limits the model’s direct application to real-world scenarios with fluctuating environments. Future research will therefore focus on enhancing the model’s practical utility and performance. This will involve incorporating real-time environmental parameters to improve on-site adaptability, while simultaneously exploring newer machine learning architectures on larger datasets to unlock greater predictive accuracy and material insight.

## Figures and Tables

**Table 1:** Detailed statistics of the parameters.

Variables	Type	Minimum	Median	Maximum	Mean Value	Standard Deviation
Cement/kg/m^3^	Input	135	296	540	299	99.89
Fly ash/kg/m^3^	Input	0	90	200	76	64.14
Water/kg/m^3^	Input	132	181	228	176	20.67
Superplasticizer/kg/m^3^	Input	0	5.1	28.2	5.9	5.05
Fine aggregate/kg/m^3^	Input	594	779	945	779	63.89
Coarse aggregate/kg/m^3^	Input	801	1023	1140	1004	89.20
Strength/MPa	Output	8.54	31.84	79.9	33	14.08

**Table 2:** Comparison of Evaluation Metrics for Five Machine Learning Models (Point Estimate).

Models	Evaluation								
	R^2^	Score	MAE	Score	RMSE	Score	MAPE	Score	Rank
XGBoost	0.9304	2	1.8495	1	2.8937	2	6.44%	2	7
LightGBM	0.9133	3	2.5140	4	3.2285	3	8.71%	4	14
CatBoost	0.9388	1	1.9786	2	2.7131	1	5.45%	1	4
RF	0.8978	4	2.1494	3	3.5062	4	6.71%	3	14
BPNN	0.8821	5	2.9386	5	3.7657	5	10.13%	5	20

**Table 3:** Mix Proportion Data.

Number	Cement	Fly Ash	Water	Superplasticizer	Coarse Aggregate	Fine Aggregate
1	222	97	189	4.5	967	870
2	296	96	171	8.9	955	859
3	522	0	146	0	896	896
4	300	120	212	10	878	728
5	380	70	175	13.2	954	811
6	460	50	175	17.3	995	720
7	260	80	185	8.2	949	876
8	340	70	180	11.1	967	823
9	400	50	170	14.4	997	783
10	380	70	175	13.2	954	811

**Table 4:** Performance of the CatBoost Model on the External UCI Dataset.

Dataset	Sample Size	R^2^	MAE	RMSE	MAPE
UCI (Strategy A)	175	0.9014	2.9445	3.5827	11.21%
UCI (Strategy B)	311	0.7480	4.7929	6.9738	12.15%
Original Test Set	45	0.9388	1.9786	2.7131	5.45%

## Data Availability

The original contributions presented in this study are included in the article. Further inquiries can be directed to the corresponding author.

## References

[1] Reddy M.S., Dinakar P., Rao B.H. (2016). A review of the influence of source material’s oxide composition on the compressive strength of geopolymer concrete. Microporous Mesoporous Mater..

[2] Rajabi A.M., Moaf F.O. (2017). Simple empirical formula to estimate the main geomechanical parameters of preplaced aggregate concrete and conventional concrete. Constr. Build. Mater..

[3] Velay-Lizancos M., Perez-Ordoñez J.L., Martinez-Lage I., Vazquez-Burgo P. (2017). Analytical and genetic programming model of compressive strength of eco concretes by NDT according to curing temperature. Constr. Build. Mater..

[4] Yusuf M.O., Al-Sodani K.A.A., Adewumi A.A., Abdulkareem M., Alateah A.H. (2025). Strength and Microstructural Characteristics of Fly Ash—Waste Glass Powder Ternary Blended Concrete. Materials.

[5] Amini K., Vosoughi P., Ceylan H., Taylor P. (2019). Effect of mixture proportions on concrete performance. Constr. Build. Mater..

[6] Breysse D. (2012). Nondestructive evaluation of concrete strength: An historical review and a new perspective by combining NDT methods. Constr. Build. Mater..

[7] Hüsken G., Brouwers H.J.H. (2008). A new mix design concept for earth-moist concrete: A theoretical and experimental study. Cem. Concr. Res..

[8] Dinesh A., Prasad B.R. (2024). Predictive models in machine learning for strength and life cycle assessment of concrete structures. Autom. Constr..

[9] Al-Shamiri A.K., Yuan T.-F., Kim J.H. (2020). Non-Tuned Machine Learning Approach for Predicting the Compressive Strength of High-Performance Concrete. Materials.

[10] Rathnayaka M., Karunasinghe D., Gunasekara C., Wijesundara K., Lokuge W., Law D.W. (2024). Machine learning approaches to predict compressive strength of fly ash-based geopolymer concrete: A comprehensive review. Constr. Build. Mater..

[11] Golafshani E.M., Behnood A., Kim T., Ngo T., Kashani A. (2024). A framework for low-carbon mix design of recycled aggregate concrete with supplementary cementitious materials using machine learning and optimization algorithms. Structures.

[12] Mienye I.D., Sun Y. (2022). A survey of ensemble learning: Concepts, algorithms, applications, and prospects. IEEE Access.

[13] Liu Y., Liu L., Yang L., Hao L., Bao Y. (2021). Measuring distance using ultra-wideband radio technology enhanced by extreme gradient boosting decision tree (XGBoost). Autom. Constr..

[14] Chen T., Guestrin C. Xgboost: A scalable tree boosting system. Proceedings of the 22nd ACM SIGKDD International Conference on Knowledge Discovery and Data Mining.

[15] Liang R., Bayrami B. (2023). Estimation of frost durability of recycled aggregate concrete by hybridized Random Forests algorithms. Steel Compos. Struct..

[16] Bourdeau M., Zhai X.Q., Nefzaoui E., Guo X., Chatellier P. (2019). Modeling and forecasting building energy consumption: A review of data-driven techniques. Sustain. Cities Soc..

[17] Golafshani E.M., Behnood A., Arashpour M. (2020). Predicting the compressive strength of normal and High-Performance Concretes using ANN and ANFIS hybridized with Grey Wolf Optimizer. Constr. Build. Mater..

[18] Elshaarawy M.K., Alsaadawi M.M., Hamed A.K. (2024). Machine learning and interactive GUI for concrete compressive strength prediction. Sci. Rep..

[19] Sun Z., Li Y., Li Y., Su L., He W. (2024). Investigation on compressive strength of coral aggregate concrete: Hybrid machine learning models and experimental validation. J. Build. Eng..

[20] Wu Y., Pieralisi R., Sandoval F.G.B., López-Carreño R.D., Pujadas P. (2024). Optimizing pervious concrete with machine learning: Predicting permeability and compressive strength using artificial neural networks. Constr. Build. Mater..

[21] Meddage D.P.P., Fonseka I., Mohotti D., Wijesooriya K., Lee C. (2024). An explainable machine learning approach to predict the compressive strength of graphene oxide-based concrete. Constr. Build. Mater..

[22] Abdellatief M., Murali G., Dixit S. (2025). Leveraging machine learning to evaluate the effect of raw materials on the compressive strength of ultra-high-performance concrete. Results Eng..

[23] Abdellatief M., Hassan Y.M., Elnabwy M.T., Wong L.S., Chin R.J., Mo K.H. (2024). Investigation of machine learning models in predicting compressive strength for ultra-high-performance geopolymer concrete: A comparative study. Constr. Build. Mater..

[24] Kashem A., Karim R., Das P., Datta S.D., Alharthai M. (2024). Compressive strength prediction of sustainable concrete incorporating rice husk ash (RHA) using hybrid machine learning algorithms and parametric analyses. Case Stud. Constr. Mater..

[25] Alyami M., Khan M., Fawad M., Nawaz R., Hammad A.W.A., Najeh T., Gamil Y. (2024). Predictive modeling for compressive strength of 3D printed fiber-reinforced concrete using machine learning algorithms. Case Stud. Constr. Mater..

[26] Sami B.H.Z., Sami B.F.Z., Kumar P., Ahmed A.N., Amieghemen G.E., Sherif M.M., El-Shafie A. (2023). Feasibility analysis for predicting the compressive and tensile strength of concrete using machine learning algorithms. Case Stud. Constr. Mater..

[27] Abbas N., Umar T., Salih R., Akbar M., Hussain Z., Haibei X. (2023). Structural Health Monitoring of Underground Metro Tunnel by Identifying Damage Using ANN Deep Learning Auto-Encoder. Appl. Sci..

[28] Kumar R., Althaqafi E., Patro S.G.K., Simic V., Babbar A., Pamucar D., Singh S.K., Verma A. (2024). Machine and deep learning methods for concrete strength Prediction: A bibliometric and content analysis review of research trends and future directions. Appl. Soft Comput..

[29] Goodell J.W., Ben Jabeur S., Saâdaoui F., Nasir M.A. (2023). Explainable artificial intelligence modeling to forecast bitcoin prices. Int. Rev. Financ. Anal..

[30] Dolatsara H.A., Kibis E., Caglar M., Simsek S., Dag A., Dolatsara G.A., Delen D. (2022). An interpretable decision-support systems for daily cryptocurrency trading. Expert Syst. Appl..

[31] Wang Y., Andreeva G., Martin-Barragan B. (2023). Machine learning approaches to forecasting cryptocurrency volatility: Considering internal and external determinants. Int. Rev. Financ. Anal..

[32] Zhu Y., Taffese W.Z., Chen G. (2024). Enhancing FRP-concrete interface bearing capacity prediction with explainable machine learning: A feature engineering approach and SHAP analysis. Eng. Struct..

[33] Frie C., Riza Durmaz A., Eberl C. (2024). Exploration of materials fatigue influence factors using interpretable machine learning. Fatigue Fract. Eng. Mater. Struct..

[34] Yang S., Sun J., Zhifeng X. (2024). Prediction on compressive strength of recycled aggregate self-compacting concrete by machine learning method. J. Build. Eng..

[35] Jia J.-F., Chen X.-Z., Bai Y.-L., Li Y.-L., Wang Z.-H. (2022). An interpretable ensemble learning method to predict the compressive strength of concrete. Structures.

[36] De Maio U., Gaetano D., Greco F., Lonetti P., Pranno A. (2025). An adaptive cohesive interface model for fracture propagation analysis in heterogeneous media. Eng. Fract. Mech..

[37] Fan Z., Sun Y. (2021). A study on fatigue behaviors of concrete under uniaxial compression: Testing, analysis, and simulation. J. Test. Eval..

[38] Tran V.Q., Dang V.Q., Ho L.S. (2022). Evaluating compressive strength of concrete made with recycled concrete aggregates using machine learning approach. Constr. Build. Mater..

[39] Zhang W., Xu L., Shen Z., Ma B. (2022). A new approach for mechanical parameter inversion analysis of roller compacted concrete dams using modified PSO and RBFNN. Clust. Comput..

[40] Lim C.-H., Yoon Y.-S., Kim J.-H. (2004). Genetic algorithm in mix proportioning of high-performance concrete. Cem. Concr. Res..

[41] Ma K., Qiao L., Lin G., Xing G. (2024). Prediction of the shear strength of lightweight concrete beams without web reinforcement based on a machine learning model optimized by a genetic algorithm. Structures.

[42] Zhang X., Dai C., Li W., Chen Y. (2023). Prediction of compressive strength of recycled aggregate concrete using machine learning and Bayesian optimization methods. Front. Earth Sci..

[43] Ragaa A.B., Al-Neshawy F., Noureldin M. (2025). AI-based framework for concrete durability assessment using generative adversarial networks and bayesian neural networks. Constr. Build. Mater..

[44] Liu G., Sun B. (2023). Concrete compressive strength prediction using an explainable boosting machine model. Case Stud. Constr. Mater..

[45] (2019). Standard for Test Methods of Mechanical Properties of Ordinary Concrete Initially.

[46] Asteris P.G., Koopialipoor M., Armaghani D.J., Kotsonis E.A., Lourenço P.B. (2021). Prediction of cement-based mortars compressive strength using machine learning techniques. Neural Comput. Appl..

[47] Wu L., Li J., Zhang J., Wang Z., Tong J., Ding F., Li M., Feng Y., Li H. (2024). Prediction model for the compressive strength of rock based on stacking ensemble learning and shapley additive explanations. Bull. Eng. Geol. Environ..

[48] Wang H., Lin J., Guo S. (2025). Study on the Compressive Strength Predicting of Steel Fiber Reinforced Concrete Based on an Interpretable Deep Learning Method. Appl. Sci..

[49] Li Z. (2022). Extracting spatial effects from machine learning model using local interpretation method: An example of SHAP and XGBoost. Comput. Environ. Urban Syst..

[50] (2011). Specification for Mix Proportion Design of Ordinary Concrete.

